# Criminal Abortion*Read at Calvert meeting, Brazos Valley Medical Society, May 14, 1901.

**Published:** 1901-09

**Authors:** J. P. Oliver

**Affiliations:** Caldwell, Texas


### TEXAS MEDICAL JOURNAL.

ESTABLISHED JULY, 1885.

PUBLISHED MONTHLY.—SUBSCRIPTION $1.00 A YEAR.

VOL. XVII. AUSTIN, SEPTEMBER, 1901. No. 3.

### Original Contributions.

### Criminal Abortion.*

BY J. P. OLIVER, M. D., CALDWELL, TEXAS.

In the discussion of criminal abortion, it is not expected, nor do I intend to elaborate all of the causes of natural abortion, but to offer a few thoughts on one of the most heinous crimes practiced by civilized peoples, as well as savage. And I shall only deal with abortion by natural causes in order to establish the criminal side of it. The history of abortion, it has been stated, is the history of civilization, perhaps more correctly stated, the history of races, their rise and fall. Abortion from natural causes, as well as criminal, is now, and has at all times, been practiced by savage as well as civilized peoples, and develops with the progress of civilization and Christianity. Natural abortion, or that produced by the common causes of mother and child, in my experience is much less frequent among the laboring class of peoples than the wealthy and higher classes of society. Expert abortionists exist everywhere, and it would seem that in the larger cities of the United States, as well as other countries, they flourish in their nefarious work and are rarely ever convicted, perhaps on account of the secrecy of the crime. Abortion is legalized in some countries such as Japan, China and Hindoostan; in Arabia and New Caledonia on account of

*Read at Calvert meeting, Brazos Valley Medical Society, May 14, 1901.

the scarcity of food. It is also committed in the isles of the sea for the same reasons. In densely populated countries, where it is difficult to support large families, only two or three children are allowed to the family, and in some countries the women are not permitted to marry under thirty-six years of age. Under this state of social affairs there is no wonder that force and other noxious things are often resorted to for the expulsion of the fœtus. But in countries where civilization and Christianity have reached a high state it becomes more heinous from the fact that it is often committed among the educated, the refined and the wealthy. Sometimes it is the case in large cities that some poor girl has sacrificed her honor for bread for herself or for those dependent upon her for a support. There is the lady of fashion much more culpable, who cannot give up the pleasures of society for the cares of maternity; or the society belle who would resort to any and every means for the sake of retaining her beauty.

Others resort to it that their round of pleasure may not be disturbed. Authorities are not agreed in their classification of abortion, some stating that abortion proper embraces the expulsion of the fœtus any time from the first month to the end of the third month of gestation, and miscarriage the time from the end of the third month up to the first of the seventh, and any time during the two latter months, premature delivery. The generally accepted theory, however, is that the expulsion of the fœtus any time from conception up to the first of the seventh month is abortion, or all of that period before the development of the placenta, and premature delivery any time during the two last months of pregnancy, or when the placenta is fully developed. The law does not make this distinction. It is criminal all through gestation for the expulsion of the fœtus, produced either by drugs or instrumental aid, whether the woman be with child or a false conception, provided the intention is to destroy the fruit of the womb. This is a subject that every physician should study well, not only for the protection of his own character and that of his patient, "but as a law abiding citizen for the protection of society and for the enlightenment of the rising generations. The Bible injunction is not only to abstain from evil, but the very appearance of evil." Because if this measure of criminal abortion is ever corrected, it must be done largely through the united efforts of the medical profession. And here I would suggest to the younger men of the profession to beware of the woman who approaches you in full vigor of health about any derangement of her catamenial periods. Sometime you may get into trouble very unexpectedly, and for all gynecological examinations and treatments never to enter your private room with a woman for examination without a third person being present. I remember being approached by a very nice looking lady, who asked for an examination at my hands, stating that she had some trouble with her monthlies. To every appearance she was the very embodiment of health, and my suspicion was aroused at once. My reply was that if a third was admitted to witness the examination, that I would make it, otherwise I could not. To this she entered a bitter protest, but finally gave over. So I proceeded to make the examination in the presence of the third party, and found her about four and one-half or five months advanced in pregnancy. After some equivocation, she confessed the matter and asked me to take charge of her case, and help her to get rid of the tertian quid; that she would pay me well for my services. I said to her very kindly that she had approached the wrong man, that it was not only murder in the sight of God, but that the laws of the State of Texas made it a penitentiary offense to produce abortion in a woman, even with her consent, unless the mother's life was in jeopardy, and murder if the mother died. Thus the interview ended. She left the county for some time and some one performed it for her during her absence from the county. Unfortunately, there are characters in every department of science, and the medical profession, I am sorry to say, is not without them along this line. With the progress of civilization, of refinement and of knowledge, this crime, strange as it may seem, rapidly develops. It is not confined to the poor, nor to the semi-civilized peoples, but is frequent among the educated, the refined and the most wealthy. And the excuse generally offered by the latter class is that the fœtus is not a living being, forgetting or denying the physiological fact that from the very moment of conception the ovum is viable in a physiological sense depending upon its mother for future development. More than this, the civil law recognizes the fœtus in utero all through gestation and you cannot deprive it of its legal rights in the division of property. Abortion at any time from natural causes of either mother or child is usually fraught with more danger than labor at full term. How much more dangerous is it to the life of the mother when instrumental means are resorted to for its production. All physicians of experience knows that more uterine trouble is produced from abortion than perhaps all other causes combined, as from one to five and seven of all pregnant women abort. With the progress of the practice of medicine at the end of the nineteenth century criminal abortionists keep pace, and in communities where it is practiced most it is due largely to the increased numbers and the increased skill of practitioners, ready to pander to all of the whims of their degenerated customers, morally, for the sake of the dollar. The greater effort of all physicians should be to eradicate this, one of the most heinous crimes known to civilization.

Effort was made not long ago by the American Medical Association, and I hope the effort will be perpetuated by every member of the Brazos Valley Association especially, to urge the great importance of this measure upon the medical profession of Texas generally and of the United States, resulting from the earnest efforts of that honored professor of obstetrics, Hugh L. Hodge, which culminated in a report of the committee on criminal abortion in 1891, and a number of papers written upon the subject at that time. So far as my investigation has extended the laws of all the States are about the same on criminal abortion with the exception of three. All except three make the destruction of the fœtus in utero any time during the period of gestation, whether by drugs or other noxious substances, or by instrumental aid (the knitting needle or common wire are devices frequently used for this purpose) of whatever kind, a felony. The term abortion is properly limited to procuring the expulsion of the contents of the womb before the sixth month of gestation. But the law makes no such distinction in point of time. As a rule criminal abortion will probably be attempted between the third and fifth month, that is about the time when the woman first becomes convinced of her pregnancy. The law thus stated, caption 100, Sections 58 and 59, of the Pennsylvania criminal statute, reads: "§58. Every woman being with child who, with intent to procure her own miscarriage, shall unlawfully administer to herself any poison, or other noxious thing, or shall unlawfully use any instrument, or other means whatsoever, with like intent; and whoever, with the intent to produce the miscarriage of any woman, whether she be or be not with child, shall unlawfully administer, etc., shall be guilty of felony." Section 59 reads: "Whoever shall unlawfully supply or procure any poison or noxious thing, or any instrument whatsoever, knowing that the thing was intended to be unlawfully used, or employed with the intent to produce the miscarriage of any woman, whether she be or be not with child, shall be guilty of a misdemeanor, and, being convicted thereof, shall be liable at the discretion of the court to be kept in penal servitude for the term of three years, or to be imprisoned for any term not exceeding two years."

Texas laws, I am informed, are about the same; in fact, the laws of the United States, England, France and Germany are all about the same, especially in France and Germany, where the law is very strict. According to an eminent teacher of medical jurisprudence, J. L. Casper, who says that "Of all the many accused, never a one was condemned, and in no one case was the crime proven." The States of Maine, Massachusetts and New Jersey have ruled by their supreme courts, that "to cause or to attempt abortion before quickening is not to be indictable even as an assault, if done with the consent of the woman." The Pennsylvania court, however, has discarded this doctrine, and has decided that the very moment the womb is instinct with embryo life and gestation has begun the crime may be perpetrated. "The distinction alluded to with regard to quickening is allowed by an acknowledged legal authority, Wharton, Criminal Law of the United States, 537, to be at variance, not only with medical experience, but with all other principles of common law. The civil rights of an infant in utero are respected equally throughout gestation, at every stage of which process, no matter how early it may be appointed executor, is capable of taking as legatee or under a marriage settlement may take specifically as a child under a general devise and may obtain an injunction to stay waste." "When in an attempt to procure abortion there is an evident intent to produce the death of the mother, and her death does actually occur, such attempt becomes murder at the common law." "But when it is only intended to commit the misdemeanor, it becomes manslaughter." The above citations of law are sufficient to show how the law defines and punishes the criminal abortionist for his offense against the laws of the country, not to say anything about his or her offense against God and society at large. To my mind this is one of the most heinous crimes known among races, and establishes the fact, beyond contradiction, the degeneracy existing in the human heart, not to mention its moral and legal effects on society. Oftentimes abortion is in its very nature a secret crime The abortionist and his patient act in concert. The patient applies to the abortionist and submits herself to his treatment. Or the midwife and the pregnant woman arrange the former to procure and the latter to take certain drugs. Besides the persons concerned, no human ear heard what was agreed to, no human tongue can testify positively what was done.

Thus the patient becomes the accomplice of the abortionist. You all know how the law views the evidence of accomplices. The secrecy and the enormity of criminal abortion makes it the more urgent upon the medical profession, especially of this great commonwealth, to exercise all of their influence against abortion by artificial means or any other noxious thing, unless the life of the mother is in imminent peril, and then only to resort to it after matur consultation.

